# *Puerariae lobatae* Radix Alleviates Pre-Eclampsia by Remodeling Gut Microbiota and Protecting the Gut and Placental Barriers

**DOI:** 10.3390/nu14235025

**Published:** 2022-11-25

**Authors:** Liping Huang, Zhongyu Liu, Peng Wu, Xiaojing Yue, Zhuoshi Lian, Peishi He, Yarui Liu, Ruisi Zhou, Jie Zhao

**Affiliations:** 1 Department of Obstetrics and Gynecology, Nanfang Hospital, Southern Medical University, Guangzhou 510515, China; 2 NMPA Key Laboratory for Research and Evaluation of Drug Metabolism, Guangdong Provincial Key Laboratory of New Drug Screening, School of Pharmaceutical Sciences, Southern Medical University, Guangzhou 510515, China; 3 Department of Urology, Nanfang Hospital, Southern Medical University, Guangzhou 510515, China; 4 Microbiome Medicine Center, Department of Laboratory Medicine, Zhujiang Hospital, Southern Medical University, Guangzhou 510280, China; 5 TCM-Integrated Hospital, Southern Medical University, Guangzhou 510315, China

**Keywords:** *Puerariae lobatae* Radix, pre-eclampsia, gut-placenta axis, gut microbiota, fecal microflora transplantation

## Abstract

Pre-eclampsia (PE) is a serious pregnancy complication, and gut dysbiosis is an important cause of it. *Puerariae lobatae* Radix (PLR) is a medicine and food homologous species; however, its effect on PE is unclear. This study aimed to investigate the efficacy of PLR in alleviating PE and its mechanisms. We used an NG-nitro-L-arginine methyl ester (L-NAME)-induced PE mouse model to examine the efficacy of preventive and therapeutic PLR supplementation. The results showed that both PLR interventions alleviated hypertension and proteinuria, increased fetal and placental weights, and elevated the levels of VEGF and PlGF. Moreover, PLR protected the placenta from oxidative stress via activating the Nrf2/HO-1/NQO1 pathway and mitigated placental damage by increasing intestinal barrier markers (ZO-1, Occludin, and Claudin-1) expression and reducing lipopolysaccharide leakage. Notably, preventive PLR administration corrected gut dysbiosis in PE mice, as evidenced by the increased abundance and positive interactions of beneficial bacteria including *Bifidobacterium*, *Blautia*, and *Turicibacter*. Fecal microbiota transplantation confirmed that the gut microbiota partially mediated the beneficial effects of PLR on PE. Our findings revealed that modulating the gut microbiota is an effective strategy for the treatment of PE and highlighted that PLR might be used as an intestinal nutrient supplement in PE patients.

## 1. Introduction

Pre-eclampsia (PE), a pregnancy-specific disorder, is characterized by hypertension and proteinuria after 20 weeks of gestation [1,2,3]. PE affects 2% to 8% of pregnancies and causes significant maternal and perinatal morbidity and mortality [4,5]. As a malignant metabolic disease, the pathogenesis of PE is complex and multifactorial. Placental ischemia caused by inadequate spiral artery remodeling is considered a central factor in the etiology of PE [6,7]. Upon placental ischemia, the release of reactive oxygen species (ROS), reactive nitrogen species (RNS), and lipid peroxides increases rapidly, while the antioxidant defense, including superoxide dismutase (SOD) and glutathione peroxidase (GPx), is reduced [8]. This condition of oxidative stress induces the release of pro-inflammatory cytokines, anti-angiogenic factors, and apoptotic fragments in the placenta, resulting in excessive inflammatory responses and endothelial dysfunction [9,10]. Ultimately, this leads to severe vasoconstriction and glomerular endotheliosis, culminating in clinical manifestations such as hypertension and proteinuria [11,12]. Notably, emerging studies found that PE patients presented with gut dysbiosis, and this microecological disorder has been actually proved to provoke PE symptoms [13,14]. Jin et al. reported that the oral administration of *Akkermansia muciniphila* alleviated symptoms of PE in rats by modulating macrophage polarization and spiral artery remodeling in the placenta through the metabolites propionate and butyrate [15]. This suggests that intestinal microorganisms play a crucial role in the development of PE, and regulating gut microbiota may be a potential treatment strategy for PE.

*Puerariae lobatae* Radix (PLR), known as ‘Asian ginseng’, is rich in nutrients including flavonoids, starch, saponins, and amino acids [16,17]. PLR is also a medicine and food homologous herb that is widely used to treat gastrointestinal and cardiovascular diseases [18,19,20]. Notably, a recent study has confirmed the safety of PLR by feeding high doses of aqueous PLR extract to rats [21]. Therefore, it is reasonable to test PLR in the treatment of PE. Accumulating studies have indicated that PLR and its active ingredients may ameliorate obesity, type 2 diabetes mellitus, and nonalcoholic fatty liver disease by modulating the gut microbiota [22,23,24,25]. Puerarin, a kind of isoflavone in PLR, has been shown to improve intestinal barrier function through enhancing goblet cells and the mucus barrier [26]. In our previous study, it was shown that the combination of PLR and Chuanxiong Rhizoma (CXR) was an effective treatment for ischemic stroke. PLR decoction alleviated gut dysbiosis by increasing beneficial bacteria such as *Oscillospira* and *Lachnospira*, which may have further mitigated damage of the gut and brain barriers, prevented intestinal microbiota translocation, and improved the neurological function in cerebral ischemic stroke rats [27]. However, the effect of PLR on PE has not been evaluated yet.

In this study, we aimed to elucidate the efficacy of PLR in improving PE and the underlying mechanisms from the perspective of gut microecology with the hope of indicating a novel strategy to prevent and treat PE.

## 2. Materials and Methods

### 2.1. Materials and Reagents

PLR in the form of medicinal slices ready for decoction was obtained from Guangzhou Weida Company (Guangzhou, China). Puerarin (purity ≥ 99.0%), daidzin (purity ≥ 98.0%), and daidzein (purity ≥ 98.0%) were obtained from the Chengdu Must Bio-Technology Company (Chengdu, Sichuan, China). Rabbit anti-Nrf2 antibody (A1244), rabbit anti-HO-1 antibody (A1346), and rabbit anti-NQO1 antibody (A19586) were provided by ABclonal (Wuhan, China). Rabbit anti-Claudin-1 antibody (YT0942) was provided by ImmunoWay (Plano, TX, USA). Rabbit anti-Occludin antibody (ab216327) and rabbit anti-ZO-1 antibody (ab216880) were provided by Abcam (Cambridge, MA, USA). Mouse anti-β-actin antibody (RM2001) was provided by Beijing Ray Antibody Biotech (Beijing, China). Formaldehyde was obtained from Tianjin Da Mao chemical reagents factory (Tianjin, China).

### 2.2. Preparation of PLR Decoction

The preparation of PLR decoction was conducted as described previously [27]. Briefly, PLR was extracted in boiling water at a ratio of 1:10 for 1 h, and the filtrate was collected. The remaining residue was boiled with water (1:6, *w*/*v*) for 1 h. The filtrate was collected and mixed with the previous filtrate. The decoction was condensed and then quantified for puerarin, daidzin, and daidzein using ultra-performance liquid chromatography (the detailed method is shown in Supplementary Table S1). The concentrations of these three compounds were 6.7, 5.6, and 0.4 mg/mL, respectively.

### 2.3. Pre-Eclampsia Mice Model and Experimental Protocol

All animal procedures were conducted in strict compliance with the National Institute of Health Guide for the Use and Care of Laboratory Animals and approved by the Institutional Animal Ethical Care Committee of Southern Medical University Experimental Animal Center. Male and female C57BL/6 mice (8–10 weeks and weighing 19–20 g) were purchased from SPF (Beijing, China) Biotechnology Co., Ltd. (Beijing, China). The animals were housed under standard conditions of light and dark cycles (12 h:12 h, temperature 25 °C) with free access to food and water. After one week of acclimatization, the healthy male mice were mated overnight with female mice at a 1:2 ratio. Female mice with a plug were recognized as pregnant, and the day was defined as gestational day (GD) 0.

Then, pregnant mice were randomly divided into four groups: (1) pregnant control group (Control, *n* = 11); (2) L-NAME-induced PE model group (PE, *n* = 12); (3) L-NAME-induced mice received Pueraria decoction (containing 72 mg/kg puerarin) by oral gavage daily from GD9 to GD18 (PE+PLR d9, *n* = 11). (4) L-NAME-induced mice received Pueraria decoction (containing 72 mg/kg puerarin) by oral gavage daily from GD0 to GD18 (PE+PLR d18, *n* = 11). The Control and PE group mice were administered an equivalent volume of distilled water to exclude the effects of gavage.

Starting from GD9, the pregnant mice from groups 2 to 4 were given a 9-day subcutaneous injection of NG-nitro-L-arginine methyl ester (L-NAME) (HY-18729A, MedChemExpress, Monmouth Junction, NJ, USA) (125 mg/kg/d). Pregnant mice in the Control group were injected subcutaneously with saline (the same volume as L-NAME) for 9 days beginning on GD9. On GD18, the mice were sacrificed by exsanguination under anesthesia with inhaled 5% isoflurane in the room air. Tissues and serum were collected for further analysis.

### 2.4. Urine Analysis and Blood Pressure Measurement

Twenty-four-hour urine samples were collected from female pregnant mice. The mouse urinary protein (UP) ELISA Kit (MM-44287M1, MEIMIAN, Yancheng, Jiangsu, China) was employed to detect proteinuria according to the instructions from the manufacturer. Systolic blood pressure (SBP) was detected on GD0, 1, 4, 8, 10, 12, 14, 16 and 18 using a non-invasive BP-2010A instrument (Softron Biotechnology, Beijing, China).

### 2.5. Fecal Microbiota Transplantation (FMT)

The following procedure for FMT was modified based on previous studies [15]. Fecal material from PE+PLR d18 mice was collected and resuspended in saline at 100 mg/mL. After the centrifugation at 1000 rpm for 1 min, the supernatant was collected for microbiota transplantation.

A new batch of pregnant mice were randomly divided into three groups: (1) PE+PLR d18 group (*n* = 8); (2) PE group (*n* = 8); (3) FMT-recipient group (PE+PLR-FMT, *n* = 8). From GD3 to GD7, the mice from the PE+PLR-FMT group received antibiotics (vancomycin, 100 mg/kg; neomycin sulfate, 200 mg/kg; metronidazole, 200 mg/kg; and ampicillin, 200 mg/kg) intragastrically once a day for 5 days to deplete the gut microbiota. Each recipient mouse was orally gavaged with 0.2 mL of fecal material once a day for 10 days. As described above, starting from GD9, three groups of mice received a 9-day subcutaneous injection of L-NAME (125 mg/kg/d). On GD18, the mice were sacrificed under anesthesia, at which time tissues and serum were collected for further analysis.

### 2.6. Enzyme-Linked Immunosorbent Assay (ELISA)

The serum and placental levels of glutathione (GSH) and malondialdehyde (MDA) were determined by GSH (E-EL-0026c) and MDA (E-EL-0060c) ELISA kits (Elabscience, Wuhan, China) according to the manufacturer’s instructions, respectively. Serum LPS was quantified using a mouse LPS ELISA kit (MM-0634M1, MEIMIAN, Jiangsu, China) according to the manufacturer’s instructions. The serum levels of soluble fms-like tyrosine kinase-1 (sFlt-1), Placental growth factor (PlGF), and Vascular endothelial growth factor (VEGF) were determined by the sFlt-1 (MM-45637M1), PlGF (MM-0090M1), and VEGF (MM-0128M1) ELISA kits (MEIMIAN, Jiangsu, China) according to the manufacturer’s instructions, respectively. 

### 2.7. Quantitative Real-Time PCR (qRT–PCR) Assay

Total RNA was extracted from placenta and colon tissue using the EastepTM Super Total RNA Extraction Kit (Promega Corporation, Madison, WI, USA) according to the manufacturer’s instructions. The reverse transcription was performed to obtain cDNA using HiScript^®^ Q RT SuperMix for qPCR (+gDNA wiper) (Vazyme, Nanjing, China). PCRs were performed on the LightCycler480 (Roche Diagnostics International, Rotkreuz, Switzerland) using a GoTaq^®^ qPCR Master Mix Kit (Promega Corporation, Madison, WI, USA) with the resulting cDNAs. Relative quantification was calculated using the comparative 2^−ΔΔCT^ method. *Gapdh* was used as a control gene. All the primer sequences for the tested genes are listed in Supplementary Table S2.

### 2.8. Western Blotting (WB) Analysis

The placenta tissue samples were lysed in RIPA buffer containing 1% cocktail protease inhibitor (Meilunbio, Dalian, China). Then, the samples were separated by SDS-PAGE and transferred into PVDF membranes (Millipore, Billerica, MA, USA). After being blocked with 5% non-fat milk for 1 h, the membranes were incubated with various primary antibodies overnight at 4 °C, followed by incubation with horseradish peroxidase-conjugated secondary antibody for 1 h at room temperature. The membranes were scanned by a gel image processing system (FluorChem R, ProteinSimple, San Jose, CA, USA). β-actin was used as an internal control. The band intensity was assessed using ImageJ software.

### 2.9. Histological and Immunohistochemistry (IHC) Analysis

For hematoxylin and eosin (H&E) staining, the placenta of mice was fixed in 4% paraformaldehyde, embedded, and sectioned at 5 μm. Next, the placenta sections were stained with hematoxylin and eosin and analyzed under a light microscope.

Immunohistochemistry was performed as previously described [28]. Placenta tissue samples were incubated with anti-Occludin antibody to estimate placental barrier proteins. Colon tissue samples were incubated with anti-Occludin antibody, anti-ZO-1 antibody, and anti-Claudin-1 antibody to analyze the expression of Occludin, ZO-1 and Claudin-1 in the colon. The average optical densities of ZO-1, Occludin, and Claudin-1 in the colon and of Occludin in the placenta were analyzed using Image-Pro Plus 6.0 software (Media Cybernetics Inc., Bethesda, MD, USA).

### 2.10. Gut Microbiota Analysis

The feces of mice were collected in sterile microtubes, promptly frozen in liquid nitrogen, and stored in a refrigerator at −80 °C. The method for 16S rRNA gene sequencing was conducted as previously reported [27]. 16S rRNA amplicon sequencing to analyze gut microbiota was carried out by Huayin Company (Guangzhou, China). DNA was extracted using a PowerSoil DNA Isolation kit (Mobio, Carlsbad, CA, USA). The quality of the DNA was measured using a Nanodrop ND-1000 spectrophotometer (Thermo Electron Corporation, Waltham, MA, USA). PCR amplification of 16S rRNA sequences was performed using primer sets specific for V3-V4 regions. Subsequently, final PCR products were purified with the Qiaquick PCR Purification kit (Qiagen, Valencia, CA, USA). Purified samples were normalized to equal DNA concentration and sequenced using the Illumina Miseq sequencer PE250 (Illumina, SanDiego, CA, USA).

### 2.11. Statistical Analysis

Data were presented as the mean ± standard error of the mean (SEM). SPSS 26.0. was used to perform statistical analyses. For parametric variables, comparisons between multiple groups were performed using one-way ANOVA. When the variance was homogeneous, the least significant difference test was selected; otherwise, Dunnett’s T3 test was used. For nonparametric variables, the statistical significance of the differences was evaluated by the Wilcoxon rank-sum or Kruskal–Wallis test. A *p*-value < 0.05 was considered to indicate statistical significance. α-diversity indices were calculated using the Mothur 1.31.2 software. The Wilcoxon rank-sum or Kruskal–Wallis test was used to analyze the differences using the R 3.0.3 software. β-diversity was performed through QIIME (V1.80) software. The correlation between microbes was calculated by Spearman correlation with SPSS. Consideration was only given to correlation values (rho) > 0.6 and *p* < 0.05. Correlation network graphs were plotted using OmicStudio tools (https://www.omicstudio.cn/tool, accessed on 18 November 2021). PICRUSt (Phylogenetic Investigation of Communities by Reconstruction of Unobserved States) analysis was used to interpret metagenomes and predict Kyoto Encyclopedia of Genes and Genomes (KEGG) pathway abundances. The statistical analysis of KEGG pathway data was performed with STAMP v2.1.3.

## 3. Results

### 3.1. PLR Alleviated the Symptoms of PE in Mice

**Figure 1:**
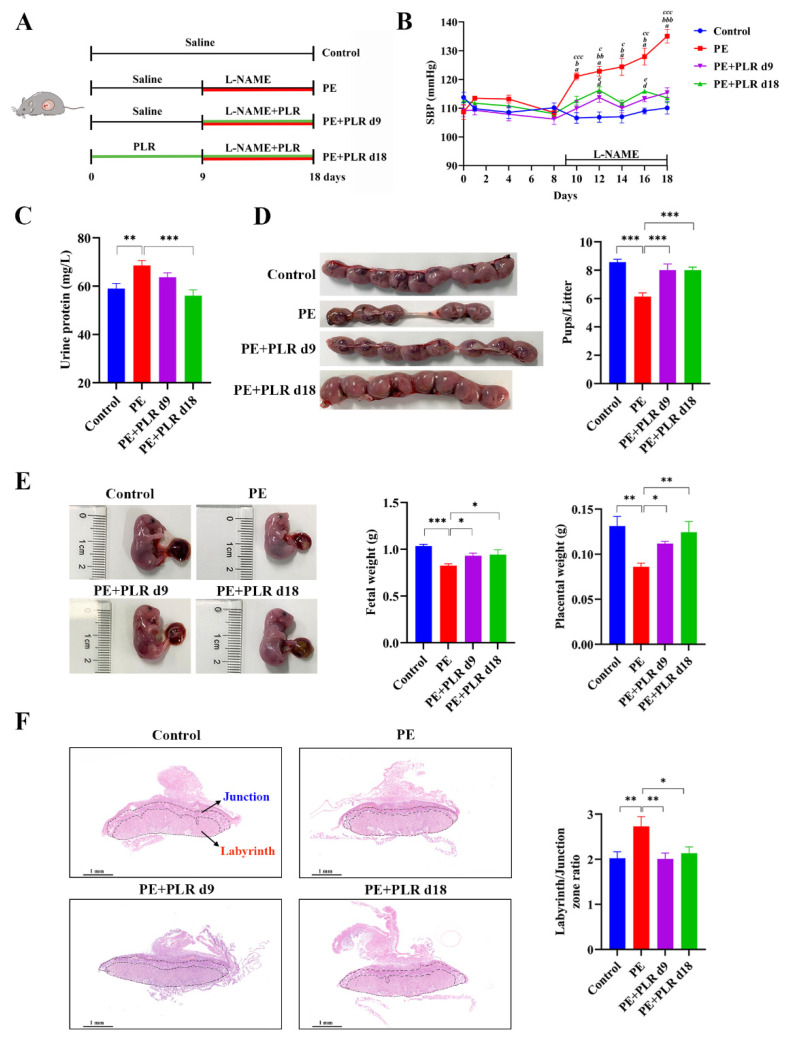
*Puerariae lobatae* Radix (PLR) alleviated pre-eclampsia (PE)-like symptoms in NG-nitro-L-arginine methyl ester (L-NAME)-induced mice. (**A**) The schematic diagram of the experimental design and procedure. (**B**) Systolic blood pressure (SBP) of the four groups was measured non-invasively. *a* indicates *p* < 0.001 for the Control group versus the PE group on the corresponding day. *b* indicates *p* < 0.05, *bb* indicates *p* < 0.01, and *bbb* indicates *p* < 0.001 for the PE+PLR d9 group versus the PE group on the corresponding day. *c* indicates *p* < 0.05, *cc* indicates *p* < 0.01, and *ccc* indicates *p* < 0.001 for the PE+PLR d18 group versus the PE group on the corresponding day. *d* indicates *p* < 0.05 for the Control group versus the PE+PLR d9 group on the corresponding day. *e* indicates *p* < 0.05 for the Control group versus the PE+PLR d18 group on the corresponding day. (**C**) The 24 h proteinuria on GD18. (**D**) Number of pups per litter. (**E**) Fetal weight and placental weight. (**F**) Representative images of H&E-stained midsagittal placental tissue sections used in histomorphological analysis (original magnification, ×25; scale bar, 1 mm). Zones are marked and indicated by labyrinth zone and junctional zone. For (**B**,**D**,**E**), *n* = 8 for each group. For (**C**), *n* = 7 for each group. For (**F**), *n* = 6 for each group. Data are expressed as the mean ± SEM. * *p* < 0.05, ** *p* < 0.01, *** *p* < 0.001 (ANOVA test).

To evaluate the efficacy of therapeutic (GD9 to GD18) and preventive (GD0 to GD18) PLR administration in alleviating PE, we used an L-NAME-induced PE mouse model (Figure 1A). As shown in Figure 1B, no significant difference in SBP was detected between each group before GD9, suggesting that PLR did not induce hypertension in the subjects of the study. However, compared with the Control group, the SBP in the PE group was significantly and persistently increased after L-NAME induction (GD10, 122.9 ± 1.2 mmHg versus 106.6 ± 1.8 mmHg, *p* < 0.001). Meanwhile, the urine protein concentration was also markedly elevated in mice of the PE group on GD18 (Figure 1C). These data indicated that the PE mouse model was successfully established.

Compared with the PE group, SBP was significantly reduced in the PE+PLR d9 and PE+PLR d18 groups on GD10. Moreover, the SBP of both PLR intervention groups remained similar to that of the Control group throughout pregnancy (Figure 1B). These results suggested that PLR effectively attenuated the SBP in PE mice. In addition, the increased level of proteinuria after L-NAME induction was significantly reduced by the preventive administration of PLR but not by therapeutic administration (Figure 1C). This indicated that preventive intervention showed a stronger ability in the improvement of proteinuria. Next, we evaluated the effect of PLR on pregnancy outcomes using the pup number, fetal weight, and placental weight as the parameters. As shown in Figure 1D, the average number of pups in the Control and PE groups was 8.6 ± 0.2 and 6.1 ± 0.3, respectively, and the number in the PE group was significantly lower than that in the Control group. However, compared with the PE group, therapeutic and preventive PLR administration significantly increased the number of pups (8.0 ± 0.5 versus 6.1 ± 0.3, *p* < 0.001; 8.1 ± 0.2 versus 6.1 ± 0.3, *p* < 0.001). Additionally, fetal weight and placental weight were markedly reduced in the PE group compared with the Control group (Figure 1E). In contrast, both PLR treatments restored placental weight and fetal weight. Compared with the PE+PLR d9 group, placental weights tended to increase in the PE+PLR d18 group, but there was no significant difference. The placenta, as a communication organ between the mother and the fetus, has nutritional, endocrine, and immunologic functions to support fetal development [29,30]. Abnormal placental development or placental dysfunction often leads to pregnancy complications such as PE [2,31]. To investigate the effect of PLR on the placenta, we next examined the structural changes in the placenta of mice in each group. As shown in Figure 1F, the ratio between the labyrinth and junctional zones was increased in placentas from the PE group compared with those from the Control group. However, the elevated ratio was reversed after both PLR interventions. In addition, the cells in the junctional zone of the PE group were disordered, and some presented necrosis. In the Control, PE+PLR d9, and PE+PLR d18 groups, the cells were intact and arranged regularly, with no obvious abnormality (Supplementary Figure S1).

The above data indicated that L-NAME-induced hypertension, proteinuria, adverse pregnancy outcomes, and placental histological lesions were ameliorated after the therapeutic and preventive administration of PLR. Moreover, the preventive intervention has an advantage over the therapeutic one in improving proteinuria and increasing placental weight.

### 3.2. PLR Ameliorated Angiogenic Imbalance in PE Mice

**Figure 2:**
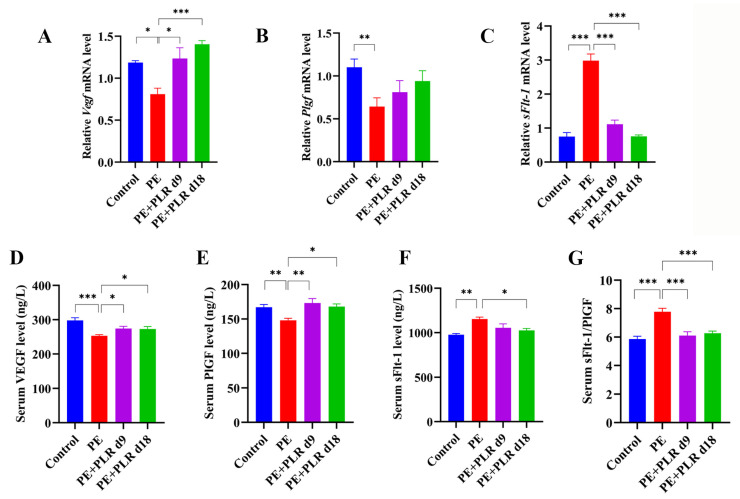
PLR reversed the angiogenic imbalance in PE mice. (**A**–**C**) Relative mRNA levels of *Vegf*, *Plgf*, and *sFlt-1* in placenta. (**D**–**F**) Serum levels of VEGF, PlGF, and sFlt-1 were measured by ELISA. (**G**) The ratio of sFlt-1 and PlGF was calculated using ELISA results. For (**A**–**C**), *n* = 6 for each group. For (**D**–**G**), *n* = 8 for each group. Data are expressed as the mean ± SEM. * *p* < 0.05, ** *p* < 0.01, *** *p* < 0.001 (ANOVA test).

Abnormalities in placentation and spiral artery invasion can lead to ischemia [32]. Ischemic placenta releases anti-angiogenic factors (soluble fms-like tyrosine kinase-1, sFlt-1) and decreases placental pro-angiogenic factors such as vascular endothelial growth factor (VEGF) and placental growth factor (PlGF), leading to angiogenic imbalance and endothelial dysfunction [33,34,35]. Therefore, we further detected the changes in angiogenic factors in PE mice. The ELISA results showed that, compared with the Control group, the serum sFlt-1 level was significantly increased in the PE group, while the serum VEGF and PlGF levels were dramatically decreased (Figure 2D–F). Moreover, we found that the ratio of sFlt-1/PlGF in the PE group was about 1.5-fold higher than that in the Control group, and a statistical difference was observed (Figure 2G). After therapeutic and preventive PLR administration, serum VEGF and PlGF levels were significantly increased, and the sFlt-1/PlGF ratio was markedly reduced (Figure 2D,E,G). In particular, preventive administration inhibited the increase in the serum sFlt-1 level better than therapeutic administration (Figure 2F). These data suggested that PLR reversed the imbalance of circulating angiogenic factors in PE mice. In addition, changes in the gene expression of *Vegf*, *Plgf*, and *sFlt-1* in the placenta were also analyzed. Consistent with the ELISA results, *sFlt-1* was markedly up-regulated in the placenta of the PE group compared with the Control group, while *Vegf* and *Plgf* were significantly down-regulated (Figure 2A–C). However, both the therapeutic and preventive administration of PLR significantly decreased *sFlt-1* and markedly increased *Vegf* (Figure 2A,C). The expression of *Plgf* in the PE+PLR d9 and PE+PLR d18 groups tended to increase compared with the PE group, although no statistical difference was observed (Figure 2B). Collectively, PLR rescued abnormal angiogenic status in PE mice.

### 3.3. PLR Attenuated Oxidative Stress and Activated the Placental Nrf2/HO-1/NQO1 Pathway in PE Mice

**Figure 3:**
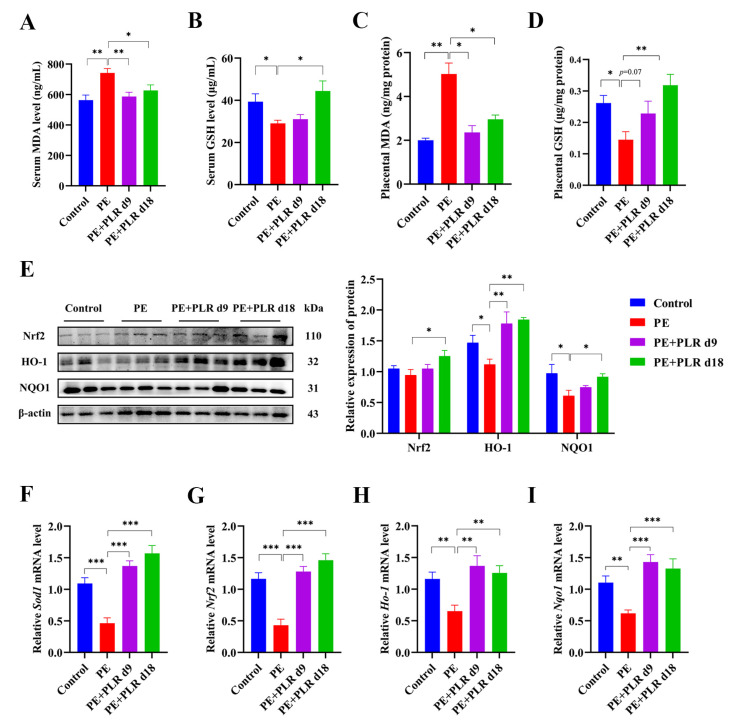
PLR attenuated oxidative stress and upregulated the placental Nrf2/HO-1/NQO1 pathway in PE mice. (**A**–**D**) ELISA was used to analyze the concentrations of malondialdehyde (MDA) and glutathione (GSH) in the placenta and serum of different groups. (**E**) Representative Western blot bands and the relative density analysis results of Nrf2, HO-1, and NQO1 in placenta. β-actin was set as a loading control, and the relative expressions were normalized to the control. (**F**–**I**) Relative mRNA levels of *Sod1*, *Nrf2*, *Ho-1*, and *Nqo1* in placenta. For (**A**–**D**), *n* = 8 for each group. For (**E**), *n* = 3 for each group. For (**F**–**I**), *n* = 6 for each group. Data are expressed as the mean ± SEM. * *p* < 0.05, ** *p* < 0.01, *** *p* < 0.001 (ANOVA test).

Increased oxidative stress has been shown to be associated with the excessive release of antiangiogenic factors from the placenta [10]. Furthermore, it has been reported that placental oxidative stress is a key intermediary event in the pathology of PE [8,36]. Therefore, we investigated the effect of PLR on oxidative stress in PE mice. As shown in Figure 3A–D,F, the PE group manifested substantially upregulated serum and placental MDA levels and downregulated serum and placental GSH levels and placental *Sod1* expression. By contrast, preventive PLR intervention considerably decreased the serum and placental MDA levels, as well as enhanced serum and placental GSH levels and placental *Sod1* mRNA expression in PE mice (Figure 3A–D,F). Therapeutic PLR intervention significantly reduced serum and placental MDA levels and markedly increased *Sod1* expression compared with the PE group (Figure 3A,C,F). However, serum and placental GSH levels were not significantly different between the two groups (Figure 3B,D). These data demonstrated that the oxidative stress in the serum and placenta of PE mice was suppressed by PLR. More importantly, the preventive intervention showed a better antioxidant capacity than the therapeutic one.

Nuclear factor-erythroid 2-like 2 (Nrf2) and its downstream transcription factor Heme Oxygenase-1(HO-1) and NAD(P)H: quinone oxidoreductase 1(NQO1) have been shown to protect cells and tissues from oxidative stress [37]. To explore whether the inhibition of placental oxidative stress by PLR could activate the Nrf2/HO-1/NQO1 signaling pathway, we determined the mRNA and protein levels of Nrf2, HO-1, and NQO1 in placenta. As shown in Figure 3G–I, the expression of *Nrf2*, *Ho-1*, and *Nqo1* was considerably reduced in the PE group compared to that in the Control group. However, the expression was remarkably increased by therapeutic and preventive PLR administration. In addition, L-NAME treatment led to the downregulation of Nrf2, HO-1, and NQO1 proteins, but a significant upregulation was observed in the mice of the PE+PLR d18 group (Figure 3E). The levels of these proteins, except for HO-1, in the PE+PLR d9 group remained unchanged relative to the PE group (Figure 3E). These data suggested that PLR could ameliorate the placental oxidative stress by activating the Nrf2/HO-1/NQO1 pathway.

### 3.4. PLR Alleviated Intestinal and Placental Inflammation and Barrier Injury in PE Mice

**Figure 4:**
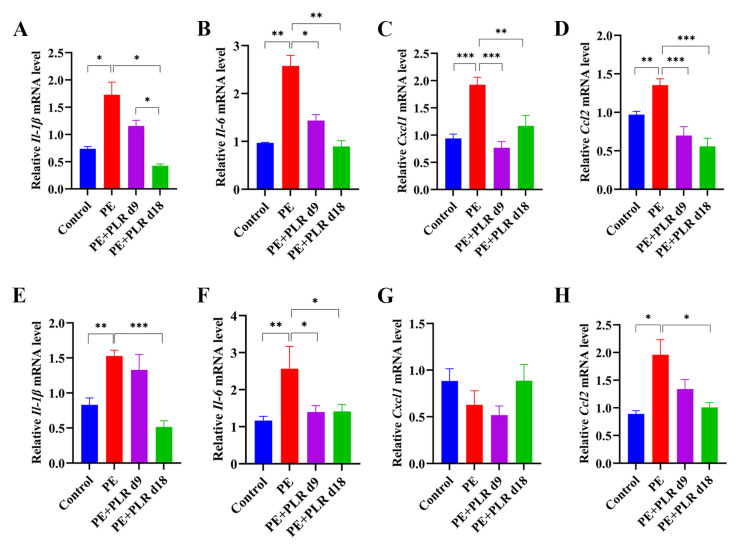
PLR attenuated gut and placental inflammation in PE mice. (**A**–**D**) Relative mRNA levels of interleukin-1β (*Il-1β*), interleukin-6 (*Il-6*), C-X-C motif chemokine ligand 1 (*Cxcl1*), and C-C motif chemokine ligand 2 (*Ccl2*) in placenta. (**E**–**H**) Relative mRNA levels of *Il-1β*, *Il-6*, *Cxcl1*, and *Ccl2* in the colon. In this figure, *n* = 6 for each group. Data are expressed as the mean ± SEM. * *p* < 0.05, ** *p* < 0.01, *** *p* < 0.001 (ANOVA test).

**Figure 5:**
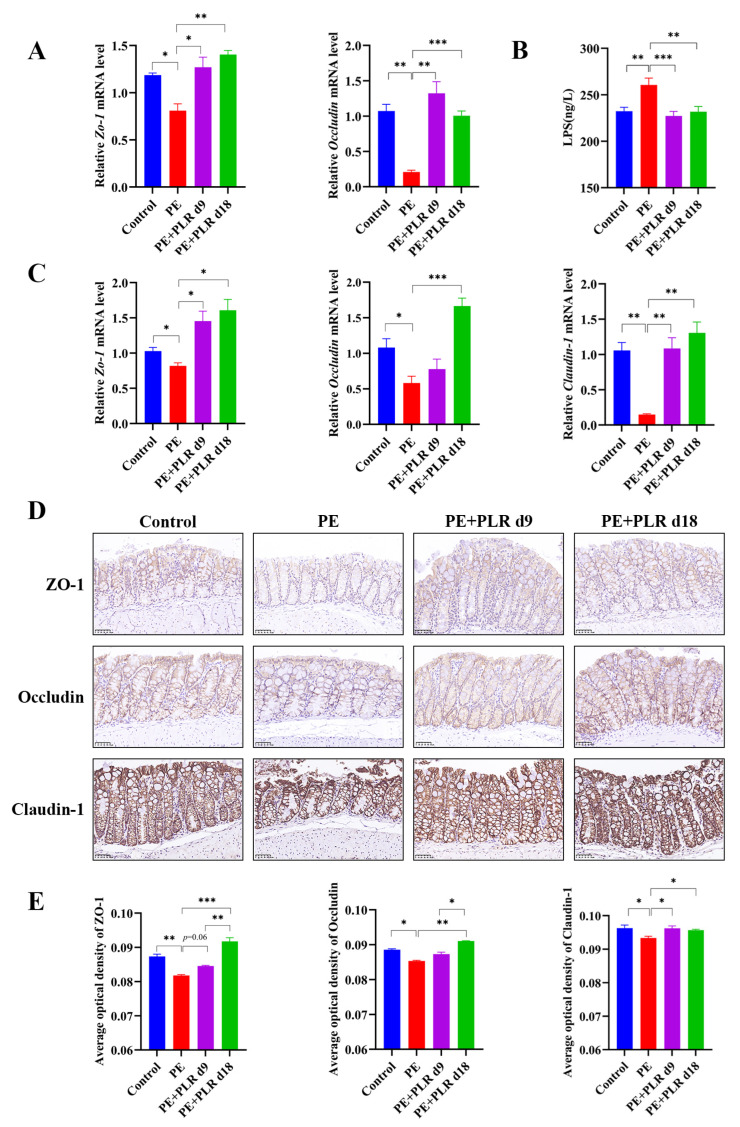
PLR protected the gut and placental barriers in PE mice. (**A**) Relative mRNA levels of *Zo-1* and *Occludin* in placenta. (**B**) Serum lipopolysaccharide (LPS) levels were measured by ELISA. (**C**) Relative mRNA levels of *Zo-1*, *Occludin*, and *Claudin-1* in the colon. (**D**) Representative image of ZO-1, Occludin, and Claudin-1 immunohistochemistry staining in colon tissues (original magnification, ×400; scale bar = 50 µm). The average optical densities of ZO-1, Occludin, and Claudin-1 in the colon (**E**) were measured by Image-Pro Plus software. For (**A**,**C**), *n* = 6 for each group. For (**B**), *n* = 7 for each group. For (**E**), *n* = 4 for each group. Data are expressed as the mean ± SEM. * *p* < 0.05, ** *p* < 0.01, *** *p* < 0.001 (ANOVA test).

Enhanced placental inflammatory response is one of the main features of PE [13,38,39]. Thus, we investigated the role of PLR in placental inflammation in PE mice by evaluating changes in the expression of pro-inflammatory cytokines and chemokines factors among the four groups. Significantly higher expressions of interleukin-1β (*Il-1β*), interleukin-6 (*Il-6*), C-X-C motif chemokine ligand 1 (*Cxcl1*), and C-C motif chemokine ligand 2 (*Ccl2*) were detected in the placenta of the PE group compared to the Control group, whereas therapeutic and preventive PLR administration inhibited the elevation (Figure 4A–D). These results suggested that PLR significantly inhibited the placental inflammatory response in PE mice. In addition, the release of multiple inflammatory mediators in the placenta stimulates the persistence of inflammation, leading to the disruption of tight junctions and weakening barrier function [40]. Therefore, we further analyzed the effect of PLR on the placental barrier. As shown in Figure 5A, the mRNA expression of *Zo-1* and *Occludin* was remarkably reduced in the placenta of the PE group compared to the Control group but elevated in the PE+PLR d9 and PE+PLR d18 groups. Furthermore, immunohistochemical results showed that the substantial loss of Occludin induced by L-NAME treatment was considerably restored in mice administered with PLR (Figure S2). These data revealed that PLR ameliorated placental barrier injury in PE mice. 

Studies have shown that maternal intestinal damage may lead to the translocation of gut bacteria or bacterial products, which may ultimately contribute to abnormalities in placental development and function [41]. Consequently, we next investigated whether PLR affected the gut. We found that the mRNA levels of *Il-1β*, *Il-6*, and *Ccl2* in the PE group were significantly elevated compared with those in the Control group, which was reversed by preventive PLR administration (Figure 4E–H). Compared with the PE group, the expression of *Il-6* was significantly decreased in the PE+PLR d9 group, and the expression of *Il-1β*, *Cxcl1*, and *Ccl2* also showed a downward trend (Figure 4E–H). These data suggested that PE mice had obvious intestinal inflammation, which could be attenuated by PLR administration. Moreover, we further found that there were obviously elevated serum LPS levels in the PE group compared to the Control group (Figure 5B). Notably, both the therapeutic and preventive supplementation of PLR significantly decreased serum LPS content compared to the PE group. This suggested that PLR reduced gut permeability and LPS leakage. To further confirm the beneficial effect of PLR in improving intestinal barrier function, we analyzed the expression levels of the tight junction proteins among the four groups. Immunohistochemical analysis illustrated that the levels of ZO-1, Occludin, and Claudin-1 were significantly decreased in the colon of the PE group compared to the Control group (Figure 5D,E). However, therapeutic and preventive PLR administration markedly restored the loss of tight junction proteins (Figure 5D,E). Meanwhile, the protein expression of ZO-1 and Occludin in the PE+PLR d18 group was higher than that in the PE+PLR d9 group. As expected, mRNA expression, determined by qPCR, was also similar to the previous results (Figure 5C). These data suggested that PLR induced gut barrier reinforcement. More importantly, the preventive intervention of PLR performed better than therapeutic intervention in promoting the expression of tight junction proteins.

### 3.5. PLR Remodeled Intestinal Microbiota in PE Mice

**Figure 6:**
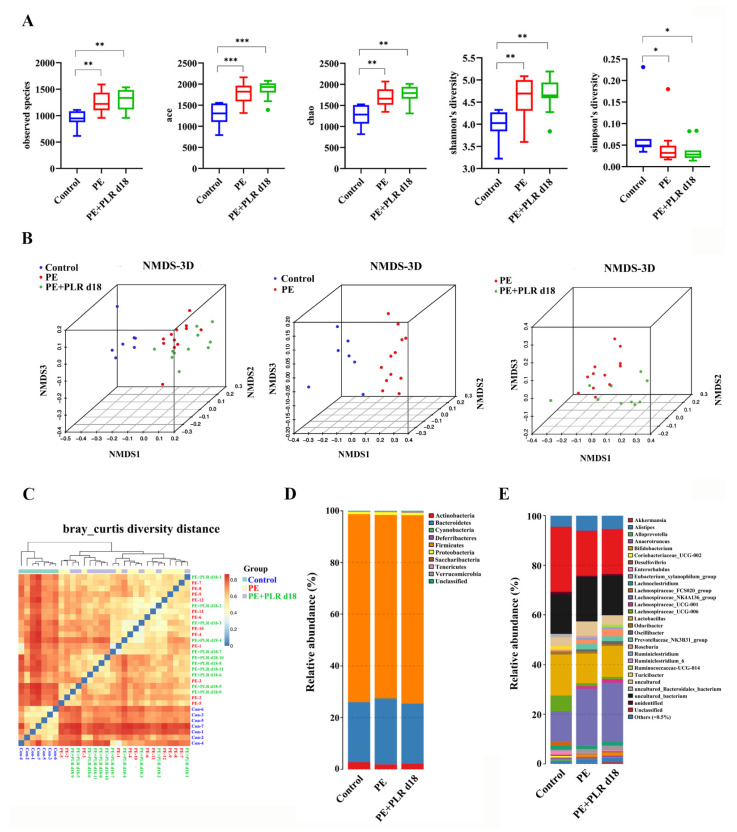
PLR affected the composition of gut microbiota in PE mice. (**A**) α-Diversity analysis including the observed species, Ace, Chao, Shannon, and Simpson indexes. β-Diversity analysis including the nonmetric multidimensional scaling (NMDS) (**B**) and heatmap (**C**) based on the Bray–Curtis diversity distance. (**D**,**E**) Relative abundance at the phylum level and genus level. In this figure, *n* = 7–12 for each group. Data are expressed as the mean ± SEM. * *p* < 0.05, ** *p* < 0.01, *** *p* < 0.001 (Kruskal–Wallis test).

Clinically, patients with PE presented gut microbiota dysbiosis [13]. Increasing studies have demonstrated that improving gut dysbiosis may be an effective approach to alleviating PE [42,43,44]. Therefore, 16S rRNA gene sequencing was employed to investigate whether PLR affected the composition of intestinal microbiota in PE mice. Considering that the preventive intervention of PLR was more effective than the therapeutic one in alleviating PE, the gut microbiota of the PE+PLR d18 group was chosen for the following analysis. First, the α-Diversity analysis was conducted to evaluate the richness and diversity of bacterial species. We found that observed species, Ace, Chao, and Shannon indexes were significantly increased and the Simpson index was markedly decreased in the PE group and PE+PLR d18 group compared with those in the Control group (Figure 6A). Moreover, β-Diversity analysis based on the Bray–Curtis diversity distance was used to measure the overall structure difference among the Control, PE, and PE+PLR d18 groups. Our results demonstrated that the Control and PE groups were clearly distinguished on the nonmetric multidimensional scaling (NMDS) plot and beta diversity heatmap, indicating that the composition of the gut microbiota was evidently altered in the mice of the PE group (Figure 6B,C). Meanwhile, the difference between the PE+PLR d18 and PE groups was also clearly displayed on the NMDS plots. These data suggested that the PLR altered the structure of the gut microbiota following the PE.

**Figure 7:**
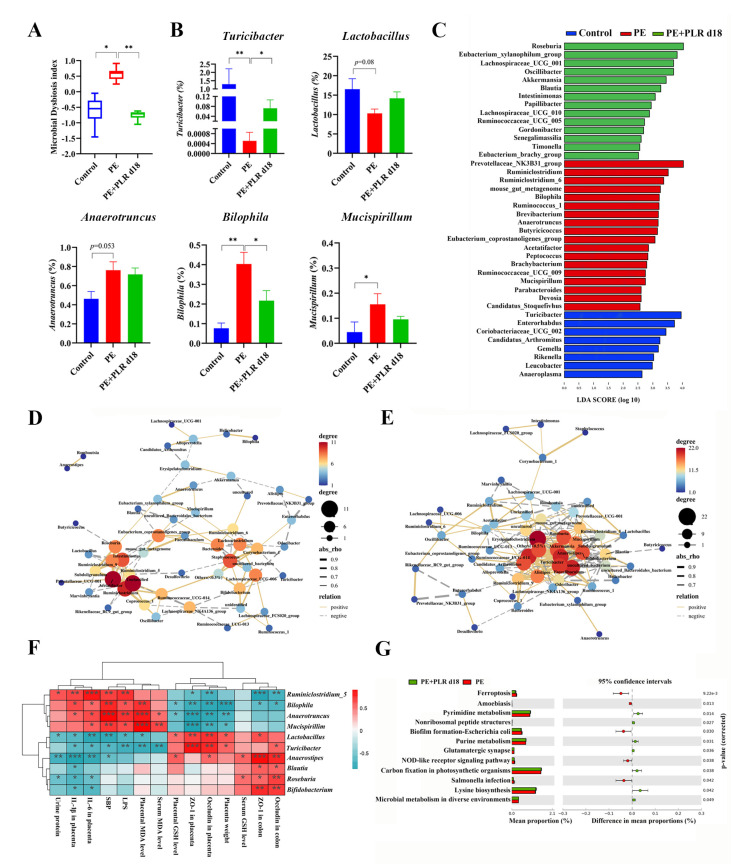
PLR corrected intestinal dysbacteriosis in PE mice. (**A**) Microbial Dysbiosis index for each group. (**B**) Comparison of the gut microbiota of mice among the three groups by *Turicibacter*, *Lactobacillus*, *Anaerotruncus*, *Bilophila*, and *Mucispirillum*. (**C**) Biomarkers at the genus level for each group screened based on LDA score. Different colors represent different groups. (**D**,**E**) Correlation network of gut microbiota in the PE and PE+PLR d18 groups. (**F**) Correlation heatmap of differentially abundant microbial genera and representative indicators. Correlations were determined by calculating Spearman’s correlation coefficient. (**G**) Pathways that were predicted to show significantly different abundances between the PE group and the PE+PLR d18 group according to the Kyoto Encyclopedia of Genes and Genome (KEGG) pathway analysis. In this figure, *n* = 7–12 for each group. Data are expressed as the mean ± SEM. * *p* < 0.05, ** *p* < 0.01, *** *p* < 0.001 (Kruskal–Wallis test).

To further explore how the microbiota structure changed, the relative abundances of the predominant phyla and genus were compared across the three groups. As shown in Figure 6D, at the phylum level, *Bacteroidetes*, *Actinobacteria*, and *Firmicutes* were the major phyla among the three groups. Compared with that in the Control group, the relative abundances of *Cyanobacteria* and *Deferribacteres* were increased in the PE group, while the abundances of *Actinobacteria* and *Firmicutes* were decreased. Treatment with PLR increased the relative abundance of *Actinobacteria* and *Verrucomicrobia* and reduced the abundance of *Saccharibacteria*. The genus level further reflected the relative differences in gut microbiota composition and abundance, and we selected 12 genera and utilized bar charts to display their detailed information (Figure 6E, Figure 7B and Figure S3). Compared to the Control group, *Ruminiclostridium_6*, *Bilophila*, and *Mucispirillum* showed significantly elevated relative abundances, whereas *Turicibacter, Coprococcus_1*, and *Lactobacillus* had decreased abundances in the PE group. However, PLR treatment remarkably reduced the abundances of *Bilophila* and *Ruminiclostridium_6*, while increased relative abundances of *Turicibacter*, *Blautia*, *Faecalibaculum*, *Bifidobacterium*, and *Anaerostipes* were witnessed in the PE+PLR d18 group.

Next, linear discriminant analysis (LDA) effect size (LEfSe) was used to obtain species with the most significant differences in abundance among the groups. As shown in Figure 7C, *Ruminiclostridium*, *Ruminiclostridium_6*, *Bilophila*, *Ruminococcus_1*, and *Anaerotruncus* were more abundant in the PE group. The levels of *Roseburia*, *Akkermansia*, *Blautia*, and *Papillibacter* were increased in the PE+PLR d18 group compared to the PE group (Figure 7C). Meanwhile, *Roseburia* was the species with the most significant differences in abundance in the PE+PLR d18 group. The above results suggested that PLR obviously altered the composition of the gut microbiota in PE mice, including reducing pathogenic bacteria and enriching beneficial bacteria. Next, we combined 10 of the most relevant taxa that characterized each group of mice and calculated the microbial dysbiosis index (MDI). The MD index value was significantly higher in the PE group compared to the Control group, which was reversed by PLR administration (Figure 7A).

Inter-bacterial interactions are important for the stability and composition of the gut micro-ecosystem [45]. After analyzing of the Spearman correlation among microbiota, the relationship and interaction among gut microbiota were determined. In the PE group, a significant negative correlation was seen between *Corynebacterium_1* and *Turicibacter*, as well as between *Alistipes and Akkermansia* (Figure 7D). Moreover, there was a significant positive correlation between *Bilophila* and *Helicobacter* and between *Anaerotruncus* and *Mucispirillum* (Figure 7D). However, PLR intervention greatly changed bacterial interactions in PE mice, such as reducing the positive correlation between *Bilophila* and *Helicobacter*. The significant positive correlation between *Anaerotruncus* and *Mucispirillum* was lost after PLR treatment (Figure 7E). It is also worth noting that PLR supplementation enabled *Bifidobacterium* to establish positive interactions with *Lactobacillus*, *Roseburia*, and *Turicibacter*; *Turicibacter* also established positive interactions with *Akkermansia* and *Roseburia*. Furthermore, *Bifidobacterium* and *Turicibacter* had a significantly negative correlation with *Mucispirillum* after PLR treatment (Figure 7E). These results suggested that PLR may remodel the interaction networks among the gut microbiota.

In addition, Spearman’s correlation coefficient was calculated to evaluate the potential relationship between altered intestinal genera and key indicators. As shown in Figure 7F, the abundances of *Mucispirillum*, *Anaerotruncus* and *Bilophila*, which were abundant in the PE group, were positively associated with SBP, urinary protein levels, and serum LPS levels and negatively correlated with the levels of tight junction proteins in the placenta and colon. In contrast, the abundances of *Turicibacter*, *Anaerostipes*, and *Lactobacillus*, which were increased in the PE+PLR d18 group, were negatively correlated with SBP, urinary protein levels, and placental inflammatory factors and positively correlated with the levels of tight junction proteins in the placenta. The abundances of *Roseburia*, *Bifidobacterium*, and *Blautia* were positively associated with the colonic tight junction proteins levels and serum GSH levels. These correlation results supported the involvement of the microbial-gut-placental axis in the development of PE and the beneficial effect of PLR on PE.

Changes in gut microbiota composition are always accompanied by alterations in physiological function. To elucidate the impact of PLR on gut bacteria function, the Kyoto Encyclopedia of Genes and Genome (KEGG) pathway analyses were used for function prediction. We found that 28 pathways were significantly different between the PE and Control groups (Supplementary Figure S4). Adipokine signaling, glyoxylate and dicarboxylate metabolism, and D-Arginine and D-ornithine metabolism were regulated to be more active in the PE group compared to the Control group, which may be associated with PE lesions (Supplementary Figure S4). However, lysine biosynthesis, pyrimidine metabolism, and purine metabolism were notably enriched in the PE+PLR d18 group compared to the PE group, suggesting that the gut microbiota undergoing PLR treatment enhanced the functions in amino acid synthesis and nucleotide metabolism (Figure 7G).

### 3.6. Transplantation of the Gut Microbiota of PLR-Treated Mice Ameliorated the Symptoms of PE Mice

**Figure 8:**
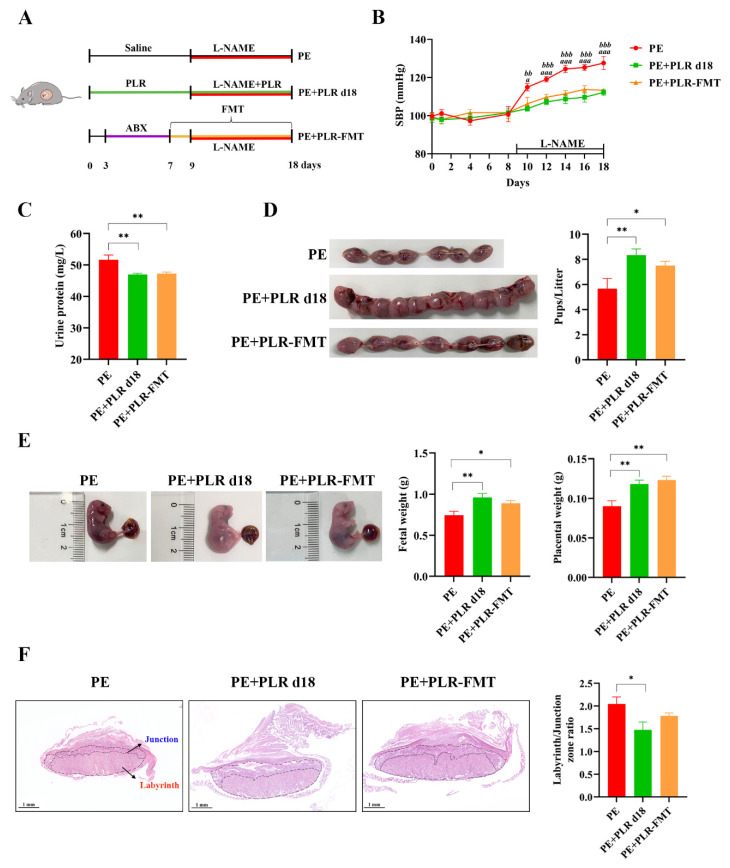
Transplantation of feces from mice treated with PLR improved symptoms of PE in mice. (**A**) The schematic diagram of the experimental design and procedure. (**B**) The SBP of three groups was measured non-invasively. *a* indicates *p* < 0.05 and *aaa* indicates *p* < 0.001 for the PE+PLR d18 group versus the PE group on the corresponding day. *bb* indicates *p* < 0.01 and *bbb* indicates *p* < 0.001 for the PE+PLR-FMT group versus the PE group on the corresponding day. (**C**) The 24 h proteinuria on GD18. (**D**) Number of pups per litter. (**E**) Fetal weight and placental weight in different groups. (**F**) Representative images of H&E-stained midsagittal placental tissue sections used in histomorphological analysis (original magnification, ×25; scale bar, 1 mm). Zones are marked and indicated by labyrinth zone and junctional zone. For (**A**–**E**), *n* = 7 for each group. For (**F**), *n* = 6 for each group. Data are expressed as the mean ± SEM. * *p* < 0.05, ** *p* < 0.01 (ANOVA test).

To investigate whether the beneficial effects of PLR on PE are mediated by the gut microbiota, a fecal microbiota transplantation experiment was performed (Figure 8A). The mice of the PE+PLR d18 group were chosen as donor mice. Our results showed that transplanting PLR-modulated microbiota significantly reduced SBP and proteinuria compared with the PE group (Figure 8B,C). Surprisingly, SBP and urinary protein content did not differ between the PE+PLR d18 group and PE+PLR-FMT groups. Furthermore, FMT restored the pup number and increased the placental and fetal weight with a similar efficacy to the donor group (Figure 8D,E). However, the ratio between the labyrinth and junctional zones in the placenta of PE mice did not change significantly after FMT intervention (Figure 8F). These results suggested that microbiota transplantation from PLR-treated mice partially alleviated PE-like symptoms in L-NAME-induced mice.

Next, we investigated the effect of PLR-regulated microbiota on angiogenic factors in PE mice. Compared with the PE group, the serum sFlt-1/PlGF ratio was significantly decreased, and the serum VEGF level was markedly increased in the PE+PLR-FMT group (Supplementary Figure S5D,G). Serum PlGF and sFlt-1 levels were not significantly different between the two groups (Supplementary Figure S5E,F). Moreover, a decreased expression of *sFlt-1* and an increased expression of *Vegf* and *Plgf* were further observed in the placenta of FMT recipient mice (Supplementary Figure S5A–C). These results showed that the transplantation of feces from mice treated with PLR restored the imbalance of anti- and pro-angiogenic factors in PE mice.

We further investigated whether the gut microbiota modulated by PLR could improve the damage of the intestine and placenta in PE mice. As shown in Supplementary Figure S6A,B, FMT significantly reduced the expressions of placental *Il-1β*, *Il-6*, and *Cxcl1* and colonic *Il-6* and *Cxcl1* in recipient mice. The above results suggested that intestinal and placental inflammation in PE mice were ameliorated after the transplantation of feces from PLR-treated mice. We further found that FMT significantly reduced serum LPS levels compared with the PE group (Supplementary Figure S7C). Moreover, an increased mRNA expression of colonic *Zo-1*, *Occludin*, and *Claudin-1* was observed in FMT recipient mice (Supplementary Figure S7D–F). Notably, the expression of placental *Occludin* was also significantly increased in the FMT group (Supplementary Figure S7B). These data suggested that transplanting PLR-modulated microbiota protected the gut and placental barriers. 

We also investigated the role of gut microbiota modulated by PLR in oxidative stress in PE mice. As shown in Supplementary Figure S7G, FMT significantly decreased the serum MDA level and markedly increased serum and placental GSH levels. In conclusion, transplanting PLR-modulated microbiota could improve the symptoms of PE, reverse angiogenic imbalance, protect the gut and placental barriers, and alleviate oxidative stress, suggesting that the gut microbiota at least partially mediated the beneficial effects of PLR on PE. 

### 3.7. FMT Attenuated the Gut Microbiota Dysbiosis of PE Mice

**Figure 9:**
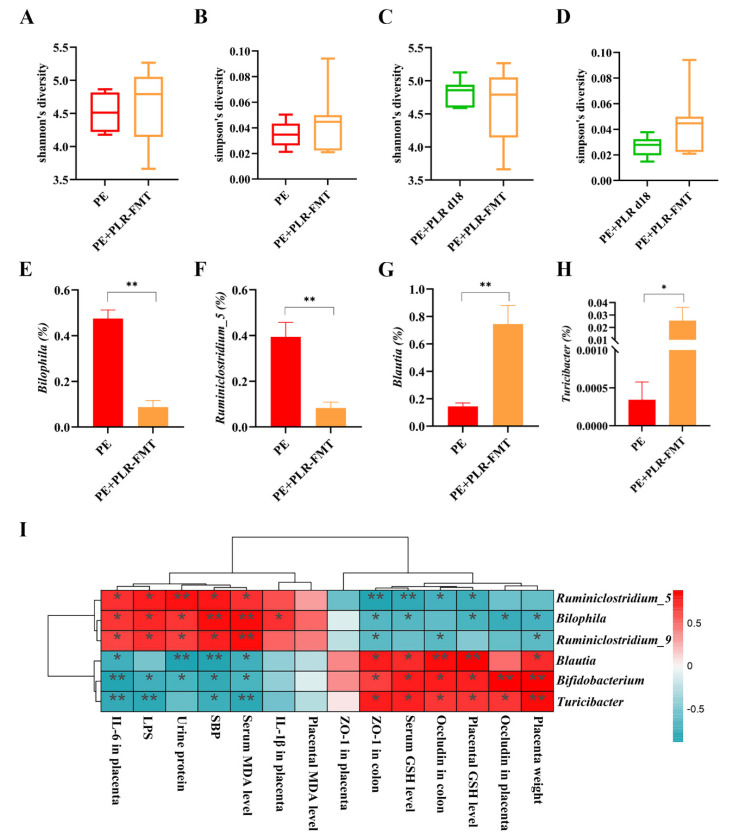
FMT altered the composition of gut microbiota in PE mice. (**A**,**B**) Comparison of α-Diversity in the PE group and the PE+PLR-FMT group. (**C**,**D**) Comparison of α-Diversity in the PE+PLR d18 group and the PE+PLR-FMT group. (**E**–**H**) Relative abundances of *Bilophila, Ruminiclostridium_5*, *Blautia*, and *Turicibacter* between the PE group and the PE+PLR-FMT group. (**I**) Correlation heatmap of differentially abundant microbial genera and representative indicators. In this figure, *n* = 6–8 for each group. Data are expressed as the mean ± SEM. * *p* < 0.05, ** *p* < 0.01 (Wilcoxon rank–sum test).

The changes in the intestinal microbiota after FMT intervention were further investigated. The α-Diversity of the PE+PLR-FMT group showed an increasing trend compared with that of the PE group, although no statistical difference was observed (Figure 9A,B). Moreover, FMT significantly reduced the relative abundances of *Bilophila*, *Ruminiclostridium_5*, and *Ruminiclostridium_9*, which were enriched in the PE group (Figure 9E,F and Figure S8A). *Blautia*, *Bifidobacterium* and *Turicibacter*, which were enriched in the donor group, were increased by FMT treatment (Figure 9G,H and Figure S8A). Notably, the interactions among microbiota were markedly altered after FMT intervention, which was consistent with the results of the PLR treatment. As shown in Figure S8B, *Turicibacter* was positively correlated with *Lactobacillus* and *Romboutsia* in FMT recipient mice, and *Lactobacillus* was positively correlated with *Anaerostipes* and *Lachnospira.* The significant positive correlation between *Staphylococcus* and *Corynebacterium_1* in PE mice was lost after FMT intervention, and *Staphylococcus* had a significant negative correlation with *Lactobacillus*. The above results suggested that FMT intervention significantly changed the gut microbiota composition and bacterial interactions in PE mice.

Subsequently, we correlated key indicators of PE with specific differential microbial taxa (Figure 9I). Spearman correlation analysis showed that the abundances of *Blautia*, *Bifidobacterium*, and *Turicibacter* were negatively correlated with SBP, urinary protein levels, LPS, and serum MDA levels and positively correlated with serum and placental GSH levels. Furthermore, KEGG pathway analysis showed that some microbiota characteristic pathways were predicted to be less active in the PE+PLR-FMT group compared to the PE group, such as bacterial chemotaxis and flagellar assembly signaling pathways. In contrast, many important metabolic pathways were regulated to be more active in the PE+PLR-FMT group, such as carbohydrate digestion and absorption and flavonoid biosynthesis (Supplementary Figure S9). These results suggested that FMT intervention attenuated bacterial virulence and pathogenicity to the host and restored beneficial substance metabolism-related pathways.

## 4. Discussion

This study was undertaken to explore whether PLR alleviates PE by modulating the microbiota-gut-placental axis. The results showed that PLR corrected gut dysbiosis in PE mice by promoting positive interactions among beneficial bacteria. Additionally, PLR strengthened the gut barrier, thereby reducing LPS leakage and ultimately protecting the placenta. The beneficial effect was also due to the activation of the placental Nrf2/HO-1/NQO1 pathway. Our findings indicate that gut microbiota interventions can be effective strategies for PE treatment and highlight that PLR may serve as an intestinal nutrient additive in PE patients.

In this study, we found that PLR ameliorated gut micro-dysbiosis caused by PE and validated that the gut microbiota was an upstream modulator of the effects of PLR. Interestingly, we observed that *Blautia* and *Turicibacter*, which were negatively correlated with SBP, urine protein, and LPS, were increased in both PLR-treated mice and FMT-treated mice. Studies have found that *Blautia*, a genus of anaerobic bacteria with probiotic properties, was significantly reduced in patients with PE [46]. Moreover, Alavi et al. reported that the genes encoding bile salt hydrolase (BSH) in *Blautia obeum* could decrease the expression of the virulent gene *tcpA* in *Vibrio cholerae*, inhibit its colonization, and relieve diarrhea in mice [47]. *Blautia* can produce bacteriocins to inhibit the growth of pathogenic bacteria, thereby maintaining homeostasis in the intestinal environment [48]. *Turicibacter*, a genus associated with glucose and lipid metabolism, has been reported to be negatively correlated with obesity, atherosclerosis, and type 2 diabetes, but its role in PE remains unclear [49,50,51]. Previous studies have shown that *Turicibacter* can produce abundant short-chain fatty acids: these increase mucin production and provide energy for intestinal epithelial cells, thus exerting a positive effect on intestinal health [52,53,54]. Therefore, we speculate that the protective effect of PLR was likely due to the enrichment of *Turicibacter* and *Blautia*, and further studies are needed to confirm this.

The gut microbiota is not a collection of many independent microorganisms but a complex ecosystem shaped by the interaction mechanisms of resource competition and nutrient symbiosis [55,56,57]. Therefore, gut microecological balance is not only defined by the composition of microbiota but also by the interactions among bacteria. Indeed, alterations of bacterial interactions may affect gut micro-ecosystem stability and further influence host health [45,55]. A clinical study revealed that the symbiosis of gut microbiota in PE patients was disordered, as evidenced by the significant increase in positive correlations among harmful bacteria and negative correlations between beneficial and harmful bacteria [15]. This disturbance of symbiotic relationships among gut microorganisms might an aspect of gut dysbiosis, which led to the development of PE. In our study, the positive correlations among harmful bacteria represented by *Bilophila* and *Helicobacter* were increased in PE mice. PLR intervention significantly weakened these correlations while enhancing the ones among beneficial bacteria including *Bifidobacterium*, *Roseburia*, and *Turicibacter*. These beneficial bacteria were negatively correlated with pathogenic bacteria in the PLR intervention group. Notably, similar alterations in microbial interactions were also observed in the FMT recipient mice. These results suggested that, by modulating the microbial interaction networks, PLR supplementation may contribute to restoring intestinal microecology, thereby improving PE. PLR contains abundant starch, which gradually absorbs water and expands during heating, breaking hydrogen bonds and rearranging into tighter crystal structures [58]. In addition, PLR is rich in cellulose, lignin, and pectin [17]. These macromolecular substances resist the hydrolysis of digestive enzymes in the small intestine and reach the colon, where they provide energy for the synergistic growth and positive interactions of specific carbohydrate-utilizing bacteria such as *Bifidobacterium, Turicibacter, Roseburia*, and *Blautia* [48,59,60,61,62,63]. Moreover, these beneficial bacteria will compete for limited nutrients and sites of epithelial adherence and produce antimicrobial factors, such as specific SCFAs and bacteriocins, that inhibit harmful bacteria [64]. This may contribute to attenuating the positive correlation among harmful bacteria and reversing gut microbiota dysbiosis. Altogether, PLR supplementation might indirectly regulate the microenvironment by affecting the interactions of some functional genera. The correlations calculated by statistics might not fully represent the true relationships among the gut microbiota, so the potential effect of PLR on the interactions of the gut microbiome strongly deserves further investigation.

Microorganisms and intestinal epithelial cells keep a mutually beneficial symbiotic relationship that contributes to maintaining intestinal homeostasis [65]. However, when the intestinal microbiota is disordered, pathogenic microorganisms will secrete virulence factors and enzymes to damage the intestinal barrier [66,67]. In our study, serum LPS levels were significantly enhanced in the PE group compared to the Control group, indicating increased intestinal permeability in PE mice. Furthermore, we found that the levels of tight junction proteins were markedly reduced in both the gut and the placenta of PE mice. Notably, PLR intervention significantly enhanced intestinal and placental defense capacity. Increasing evidence supports the presence of extensive communication between the gut and the placenta. Gohir et al. reported that an impaired intestinal barrier in mice fed a high-fat diet resulted in increased maternal circulating LPS, which may ultimately lead to hypoxia and elevated levels of inflammatory transcripts in the placenta [41]. In addition, Zhang et al. found that melatonin ameliorated placental endoplasmic reticulum stress and mitophagy in Cd-induced fetal growth restriction in mice partly via an increase in intestinal tight junction proteins [68]. Therefore, we speculate that the protective effect of PLR on the placenta may be related to the repairment of the gut barrier. Specifically, the restored intestinal barrier could inhibit pathogenic bacteria and LPS translocation, thereby attenuating placental inflammation and barrier disruption. It is also worth noting that the levels of tight junction proteins were almost restored in FMT recipient mice compared with PE mice. This result supported that gut barrier restoration partially mediates the beneficial effects of PLR on the placenta.

Studies have shown that oxidative stress can lead to premature placental aging by damaging lipids, proteins, and DNA, and it can also trigger the secretion of placental factors that result in enhanced inflammatory reactions and endothelial dysfunction, ultimately provoking PE symptoms [69,70]. It has been reported that PE patients have increased placental oxidative stress, manifested by increased lipid peroxides and the decreased expression and activity of antioxidants [71]. Furthermore, Lu et al. found that enhanced placental oxidative stress in PE patients was strongly correlated with the down-regulation of the Nrf2 pathway [72]. Nrf2 is a key transcription factor, and its activation induces the expression of downstream antioxidant genes such as HO-1 and NQO1 [73]. HO-1 can promote the degradation of heme into biliverdin, which is then converted to bilirubin by biliverdin reductase [74]. These metabolites protect cells and tissues from oxidative damage [75]. On the other hand, NQO1 is a multifunctional protein that counteracts oxidative stress by promoting the removal of ROS [76,77]. In our study, enhanced placental oxidative stress was observed in PE mice and could be reversed by PLR via rescuing the imbalance of pro- and anti-oxidative factors and activating the Nrf2/HO-1/NQO1 pathway. PLR is rich in flavonoids such as puerarin, daidzin, and daidzein, and puerarin has been reported to effectively alleviate oxidative damage by upregulating the Nrf2 pathway and increasing levels of antioxidant factors [16,20]. We speculate that, in addition to modulating the gut microbiota, the protection of the placenta by PLR may be partly attributable to the antioxidant effect of its small molecule active components. These results highlight that PLR may alleviate PE by protecting the placenta through multiple components and pathways.

In conclusion, our work suggests that PLR can ameliorate PE by reversing gut dysbiosis and protecting the gut and the placental barriers. This finding shows that gut microbiota interventions could potentially be a novel therapy for PE and demonstrates that PLR could be developed into a safe and effective intestinal nutrient supplement.

## Data Availability

The data that support the findings of this study are available from the corresponding author upon reasonable request.

## Supplementary Materials

The following supporting information can be downloaded at: https://www.mdpi.com/article/10.3390/nu14235025/s1, Table S1: Changes in the proportion of the mobile phase; Table S2: Primer sequences for qPCR; Figure S1: PLR attenuated histological lesions of the placenta in PE mice; Figure S2: PLR had a protective effect on the placental barrier in PE mice; Figure S3: PLR altered the composition of gut microbiota in PE mice; Figure S4: Pathways that are predicted to show significantly different abundances between the Control group and the PE group according to the KEGG pathway analysis; Figure S5: FMT attenuated angiogenic imbalance in PE mice; Figure S6: FMT improved colonic and placental inflammation in PE mice; Figure S7: FMT induced gut and placental barrier reinforcement and reduced oxidative stress in PE mice; Figure S8: FMT regulated the gut microbiota composition in PE mice; Figure S9: Pathways that are predicted to show significantly different abundances between the PE group and the PE+PLR-FMT group according to the KEGG pathway analysis.
