# Supplementary material for: Puerariae lobatae Radix Alleviates Pre-Eclampsia by Remodeling Gut Microbiota and Protecting the Gut and Placental Barriers
Source: Nutrients. 2022 Nov 25;14(23):5025. doi: 10.3390/nu14235025 (PMC9738998; doi:10.3390/nu14235025)
Supplement: Supplementary file 1 [file nutrients-14-05025-s001.zip › nutrients-2020653-supplementary.pdf]

# Supplementary data

**Table S1.** Changes in the proportion of the mobile phase

| Time (min) | water (%) | Acetonitrile (%) |
|---|---|---|
| 0.0 | 95 | 5 |
| 0.5 | 95 | 5 |
| 4.0 | 70 | 30 |
| 7.0 | 20 | 80 |
| 8.0 | 20 | 80 |
| 9.0 | 95 | 5 |
| 10.0 | 95 | 5 |

The column was an Agilent ZORBAX SB-C18 (3.0 × 100 mm, 1.8-Micron). The mobile phase comprised acetonitrile and water with a flow rate of 0.3 mL/min. Changes in the proportion of the mobile phase are shown in Table S1. Puerarin, daidzin, and daidzein were detected at a UV wavelength of 250 nm, 270nm and 260nm, respectively. Testosterone (240 nm) acted as the internal standard. The retention times of puerarin, daidzin, and daidzein were 2.48, 2.90, and 4.31 min, respectively.

**Table S2.** Primer sequences for qPCR.

| Gene | Forward primers (5'-3') | Reverse primers (5'-3') |
|---|---|---|
| *Il-1β* | GGGCCTCAAAGGAAAGAATC | TACCAGTTGGGGAACTCTGC |
| *Il-6* | ATCCAGTTGCCTTCTTGGGACTGA | TAAGCCTCCGACTTGTGAAGTGGT |

| | | |
|---|---|---|
| *Cxcl1* | ACCCAAACCGAAGTCATAGC | TCTCCGTTACTTGGGGACAC |
| *Ccl2* | TTAAAAACCTGGATCGGAACCAA | GCATTAGCTTCAGATTTACGGGT |
| *Zo-1* | ACGATCTCCTGACCAACGTT | GCTTTGGGTGGATGATCGTC |
| *Occludin* | ACAGTCCAATGGCCTACTCC | TACCATTGCTGCTGTACCGA |
| *Claudin-1* | GCCATCTACGAGGGACTGTG | CCCCAGCAGGATGCCAATTA |
| *Nrf2* | CCTCGCTGGAAAAAGAAGTG | CCGTCCAGGAGTTCAGAGAG |
| *Nqo1* | TTCTCTGGCCGATTCAGAGT | AGGCTGCTTGGAGCAAAATA |
| *Ho-1* | ACGCATATACCCGCTACCTG | CCAGAGTGTTCATTCGAGCA |
| *Vegf* | TTACTGCTGTACCTCCACC | ACAGGACGGCTTGAAGATG |
| *Plgf* | CGTCCTGTGTCCTTCTGAGT | CCTCCTTTCTGCCTTTGTCG |
| *sFlt-1* | GCACATGACGGAAGGAAGAC | TTCGCAGTTCAGCAGTCCTA |
| *Sod1* | CCAGTGCAGGACCTCATTTT | TTGTTTCTCATGGACCACCA |
| *Gapdh* | TGCCCCCATGTTTGTGATG | TGTGGTCATGAGCCCTTCC |

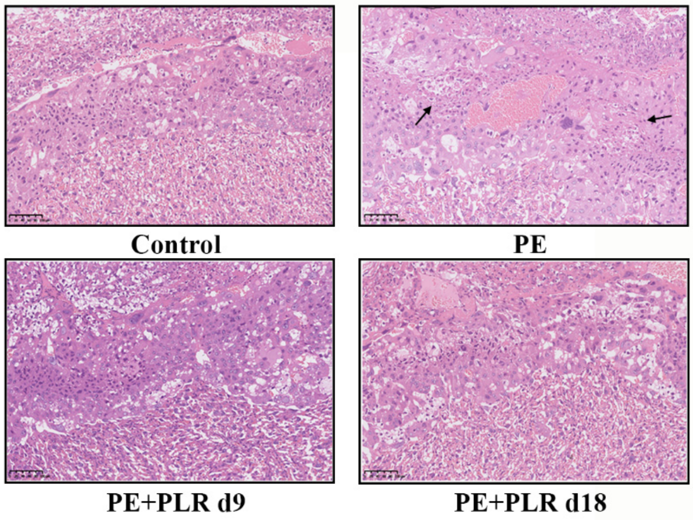


**Figure S1. PLR attenuated histological lesions of the placenta in PE mice.** Representative H&E staining of the junctional zone of mouse placentas (original magnification, ×200; scale bar, 100 μm).

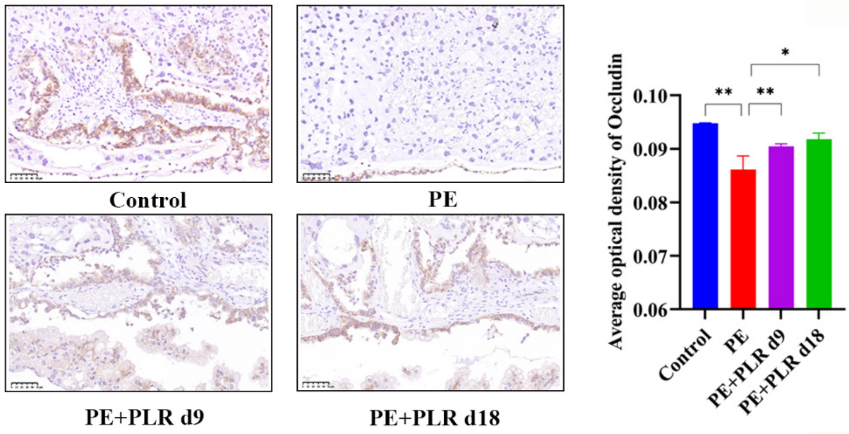


**Figure S2. PLR had a protective effect on the placental barrier in PE mice.** Representative image of Occludin immunohistochemistry staining in placental tissues (original magnification,

×400; scale bar=50 μm). And the average optical density of Occludin in placenta was measured by Image Pro-Plus software. In this figure, n = 4 for each group. Data are expressed as mean ± SEM. *p < 0.05, **p < 0.01 (ANOVA test).

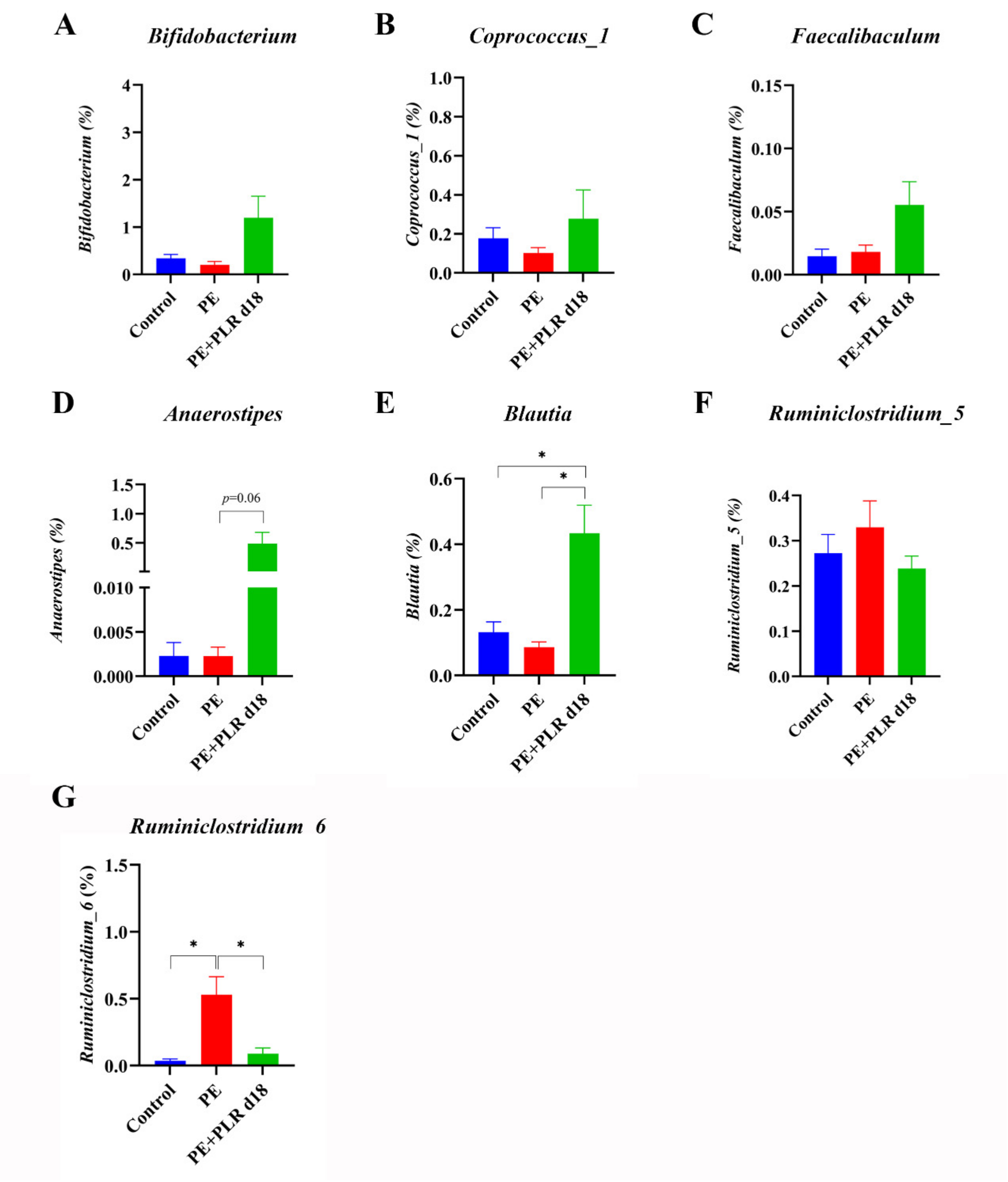


**Figure S3. PLR altered the composition of gut microbiota in PE mice.** Comparison of the gut microbiota of mice among the three groups by *Bifidobacterium* (**A**), *Coprococcus_1* (**B**),

*Faecalibaculum* (**C**), *Anaerostipes* (**D**), *Blautia* (**E**), *Ruminiclostridium_5* (**F**), and *Ruminiclostridium_6* (**G**). In this figure, n = 7-12 for each group. Data are expressed as mean ± SEM. *P<0.05 (Kruskal-Wallis test).

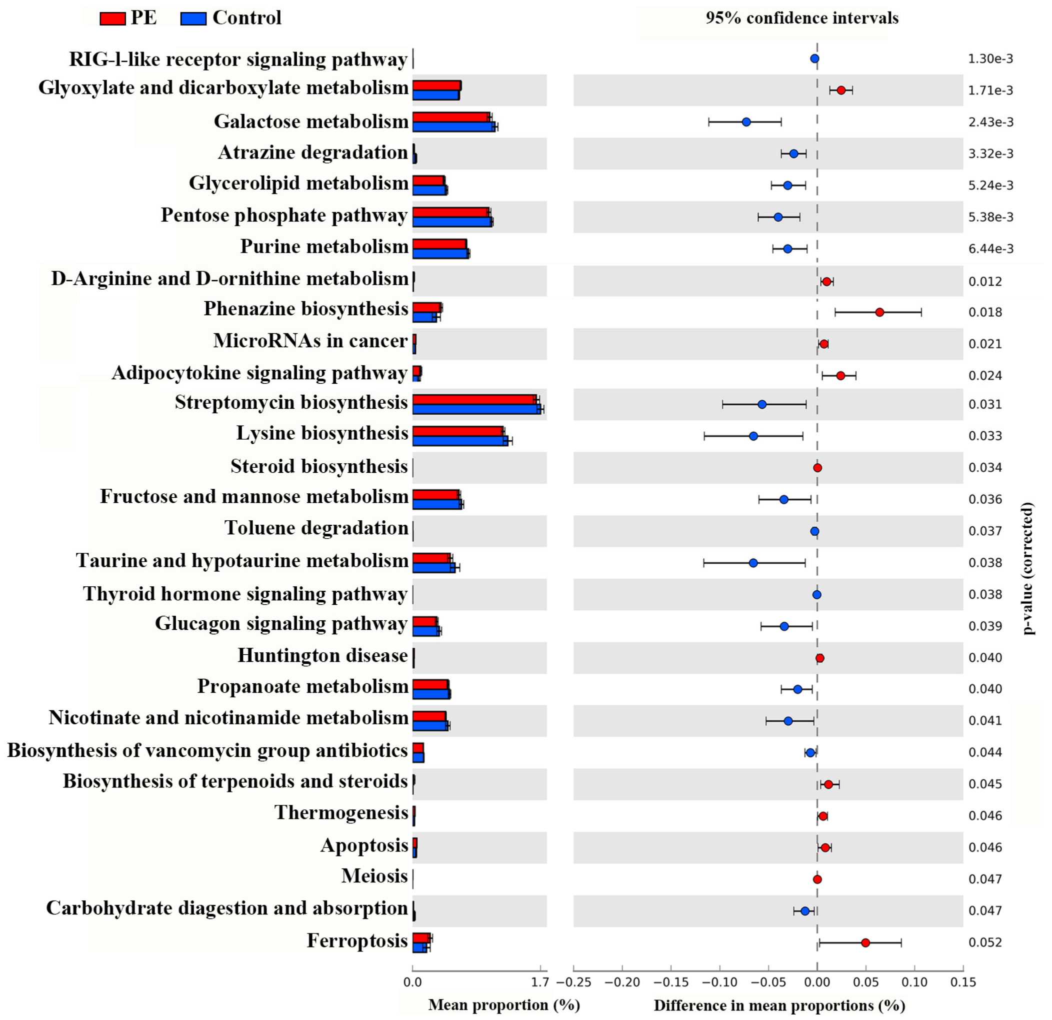


**Figure S4.** Pathways that are predicted to show significantly different abundances between the Control group and the PE group according to the Kyoto Encyclopedia of Genes and Genome (KEGG) pathway analysis.

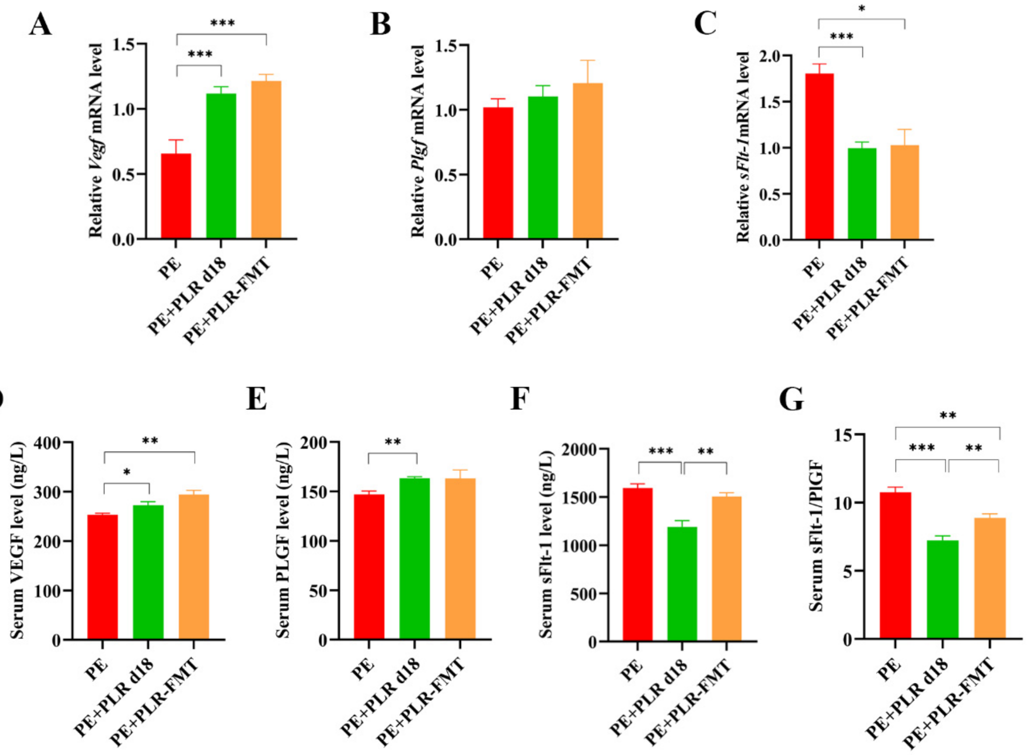


**Figure S5. FMT attenuated angiogenic imbalance in PE mice.** (**A**)-(**C**) Relative mRNA levels of *Vegf*, *Plgf*, and *sFlt-1* in placenta. (**D**)-(**F**) Serum levels of VEGF, PlGF, and sFlt-1 were measured by ELISA. (**G**) The ratio of sFlt-1 and PlGF were calculated using ELISA results. In this figure, n = 6 for each group. Data are expressed as mean ± SEM. *p < 0.05, **p < 0.01, ***p < 0.001 (ANOVA test).

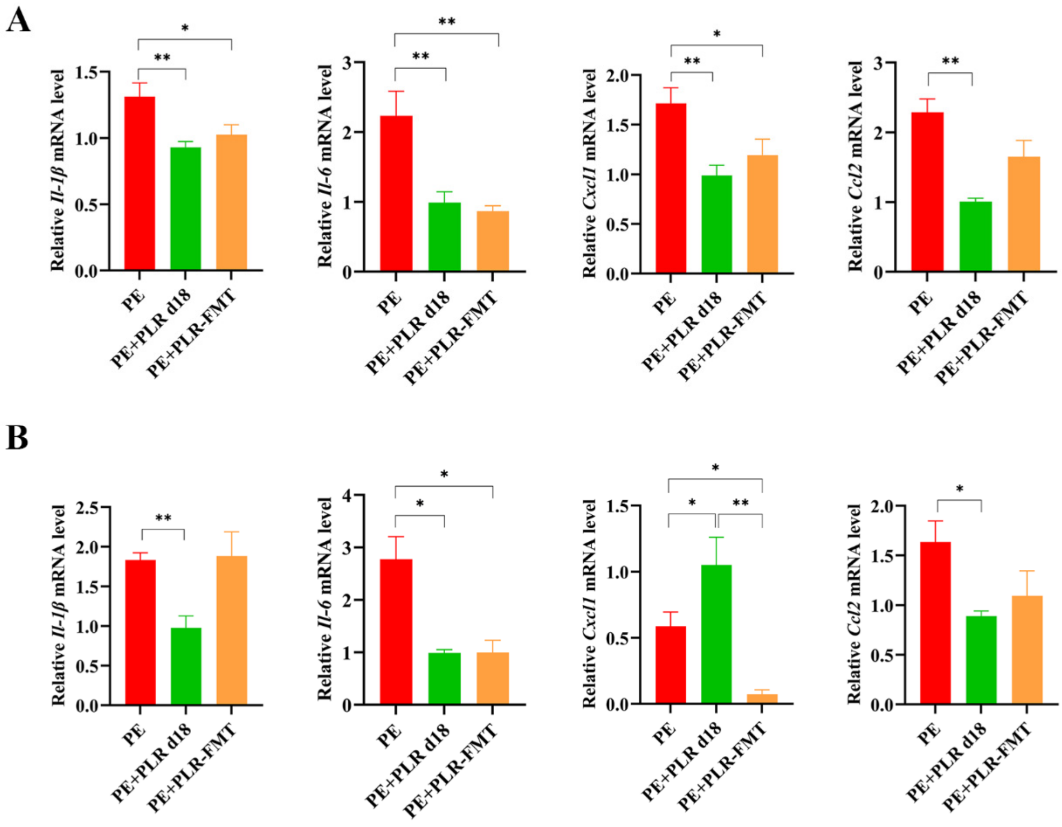


**Figure S6. FMT improved colonic and placental inflammation in PE mice.** (**A**) Relative mRNA levels of *Il-1β*, *Il-6*, *Cxcl1*, and *Ccl2* in placenta. (**B**) Relative mRNA levels of *Il-1β*, *Il-6*, *Cxcl1* and *Ccl2* in colon. In this figure, n = 6 for each group. Data are expressed as mean ± SEM. *p <0.05, **p < 0.01 (ANOVA test).

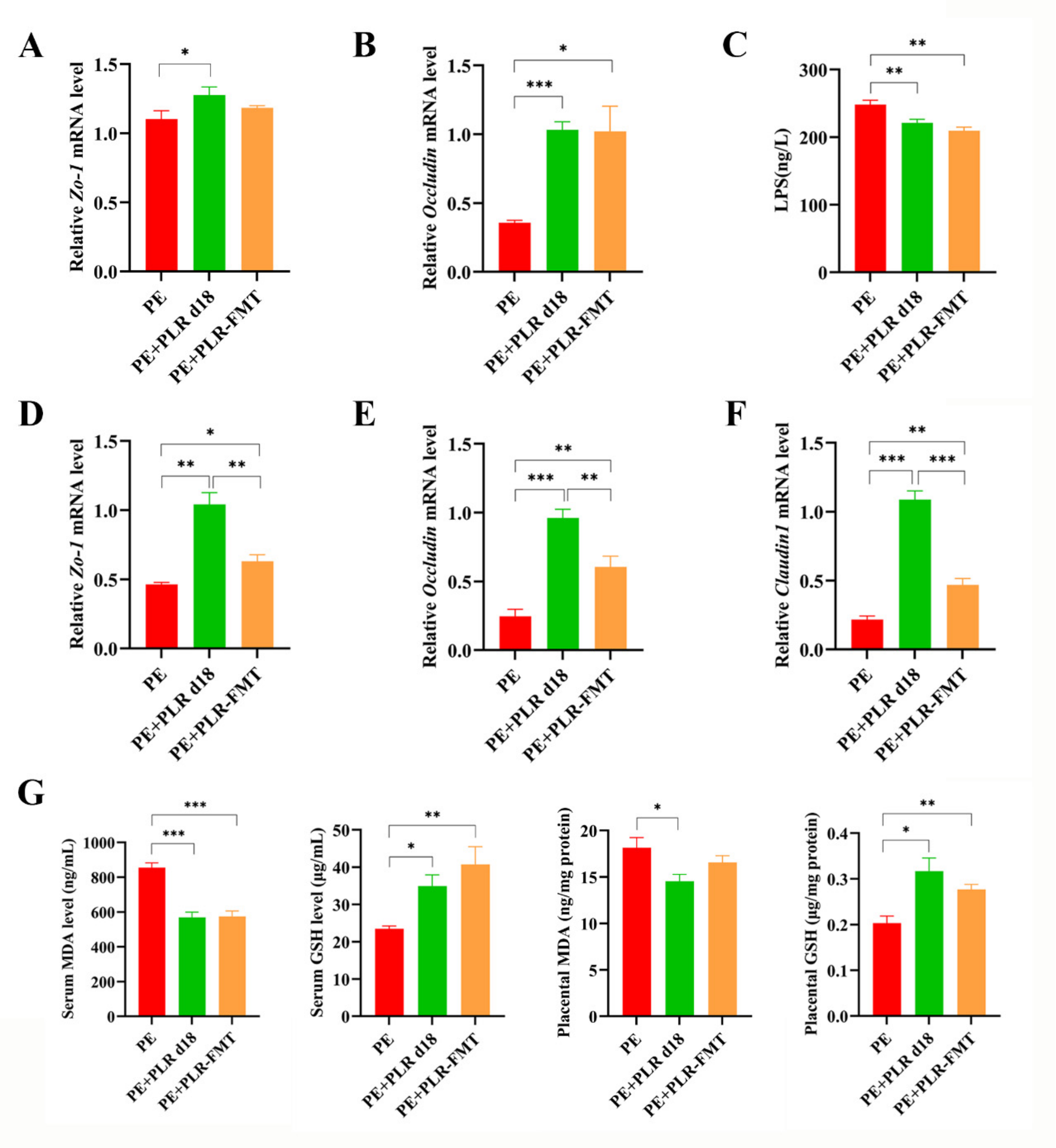


**Figure S7. FMT induced gut and placental barrier reinforcement and reduced oxidative stress in PE mice.** (**A**)-(**B**) Relative mRNA levels of *Zo-1* and *Occludin* in placenta. (**C**) Serum lipopolysaccharide (LPS) levels were measured by ELISA. (**D**)-(**F**) Relative mRNA levels of *Zo-1*, *Occludin* and *Claudin-1* in colon. (**G**) ELISA was used to analyze the concentrations of MDA and GSH in placenta and serum of different groups. In this figure, n = 6 for each group. Data are expressed as mean ± SEM. *p < 0.05, **p < 0.01, ***p < 0.001 (ANOVA test).

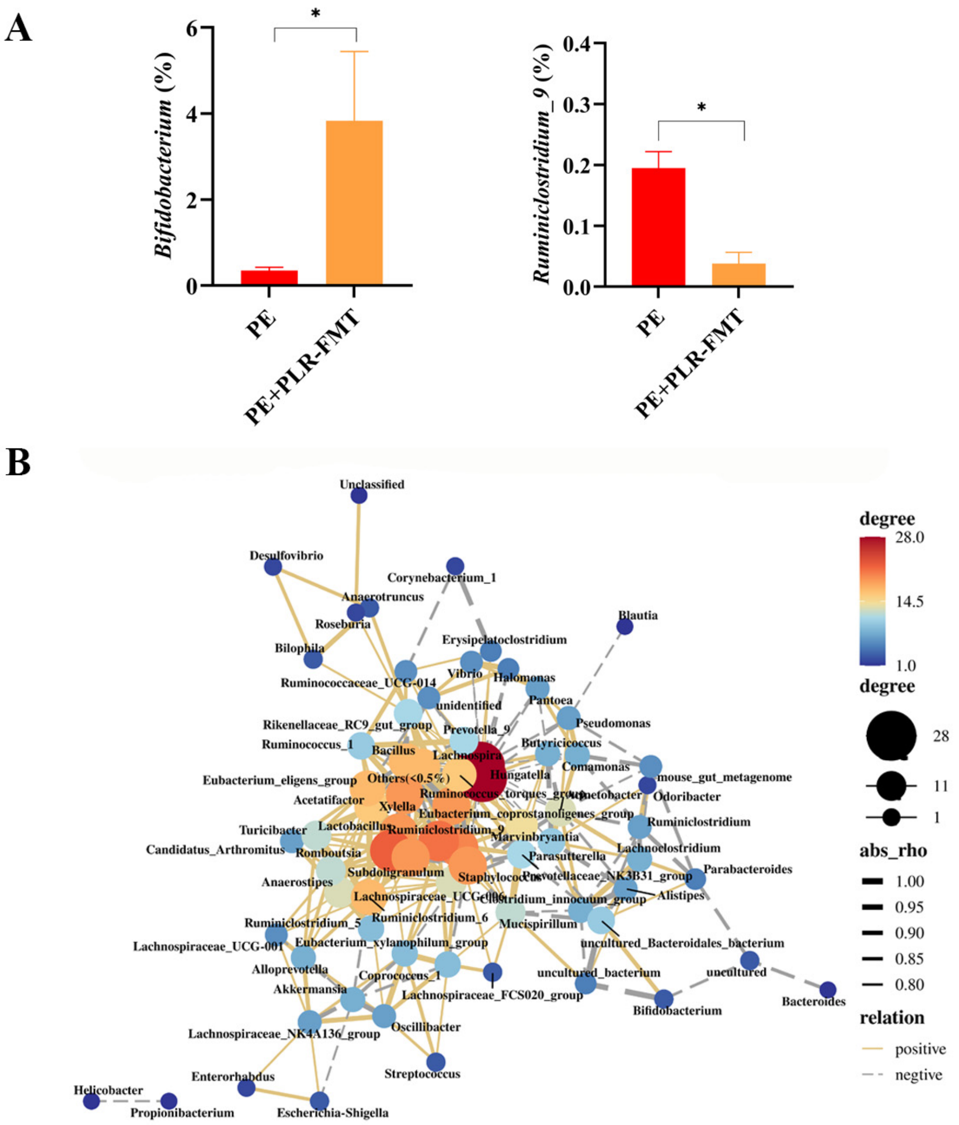


**Figure S8. FMT regulated the gut microbiota composition in PE mice.** (**A**) Relative abundances of *Bifidobacterium* and *Ruminiclostridium_9* between the PE and PE+PLR-FMT groups. (**B**) Correlation network of gut microbiota in PE+PLR-FMT group. In this figure, n = 6-8 for each group. Data are expressed as mean ± SEM. *P < 0.05 (Wilcoxon rank-sum test).

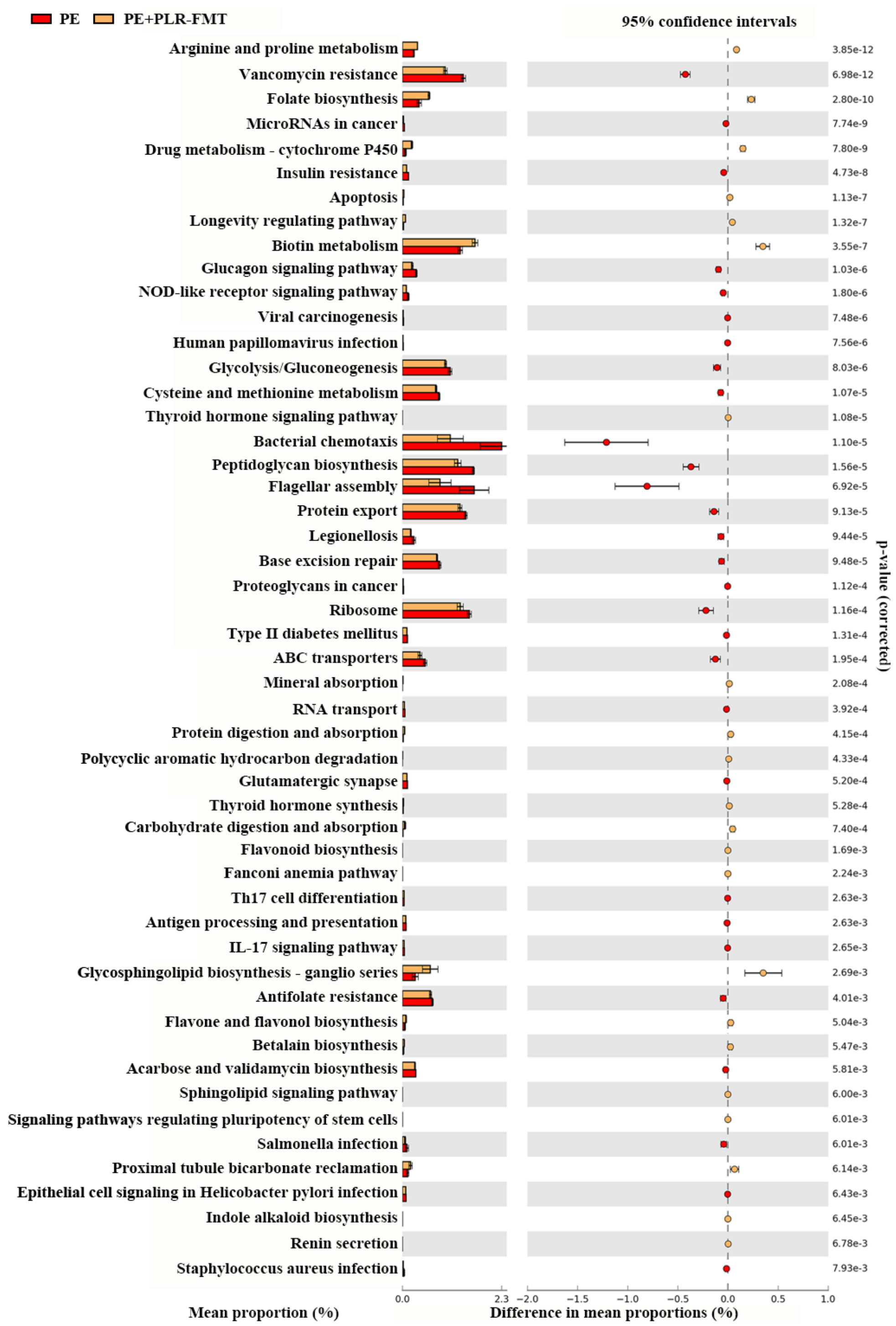


**Figure S9.** Pathways that are predicted to show significantly different abundances between the PE group and the PE+PLR-FMT group according to the KEGG pathway analysis.
